# Effect of the serum 25-hydroxyvitamin D level on risk for short-term residual dizziness after successful repositioning in benign paroxysmal positional vertigo stratified by sex and onset age

**DOI:** 10.3389/fneur.2023.1144958

**Published:** 2023-03-31

**Authors:** Jing Wu, Chun-Yan Jiang, Ying-Xia Bai, Qian Xu, Xu-Hong Sun, Hui Pan, Liang Shu, Jian-Ren Liu, Wei Chen

**Affiliations:** ^1^Department of Neurology, Shanghai Ninth People's Hospital, Shanghai Jiao Tong University School of Medicine, Shanghai, China; ^2^Department of Neurology, Huangpu Branch, Shanghai Ninth People's Hospital, Shanghai Jiao Tong University School of Medicine, Shanghai, China

**Keywords:** benign paroxysmal positional vertigo, residual dizziness, 25-hydroxyvitamin D, dizziness handicap inventory, peripheral vestibular disorder

## Abstract

**Objective:**

A low serum 25-hydroxyvitamin D (25(OH)D) level is relevant to both the occurrence and recurrence of benign paroxysmal positional vertigo (BPPV). However, whether it also contributes to residual dizziness (RD) after successful repositioning maneuvers is unknown. Therefore, this study aimed to explore the correlation between the serum 25(OH)D level and short-term RD severity in patients with BPPV after successful repositioning maneuvers.

**Methods:**

In total, 251 patients with BPPV after successful repositioning were enrolled and prospectively followed up for 1 week (W1). Serum 25(OH)D values were detected by chemiluminescence immunoassay at enrollment (W0). In addition, we explored the relationship between 25(OH)D values at baseline and RD severity at W1 in different subgroups stratified by sex and onset age (early-onset, ≤50 years; late-onset, >50 years).

**Results:**

The serum 25(OH)D level of female patients was significantly lower than that of male patients (15.9 ± 6.8 vs. 19.8 ± 6.6 ng/ml, *p* < 0.001). Its level also decreased in early-onset patients compared to late-onset ones (15.3 ± 5.9 vs. 18.0 ± 7.3 ng/ml, *p* = 0.003). In addition, early-onset female patients had lower 25(OH)D values than late-onset female patients (14.0 ± 5.5 vs. 17.1 ± 7.2 ng/ml, *p* = 0.004). However, this difference was not observed between early- and late-onset male patients. Among early-onset female patients, the 25(OH)D values of the moderate-to-severe RD group were lower than those of the minor or no RD group (10.9 ± 3.3 vs. 14.7 ± 5.7 vs. 15.0 ± 5.9 ng/ml, *p* = 0.046). Multivariate analysis found that decreased 25(OH)D values were related to the occurrence of moderate-to-severe RD in early-onset female patients (OR = 0.801; *p* = 0.022). This effect did not exist in late-onset female or male patients with BPPV.

**Conclusions:**

Age and sex differences in serum 25(OH)D levels exist in patients with BPPV. A decreased 25(OH)D level in early-onset female patients may increase the odds of moderate-to-severe RD 1 week after successful repositioning maneuvers.

## Introduction

Benign paroxysmal positional vertigo (BPPV), the most common form of peripheral vestibular disorder, is caused by the movement of otoconia in the semicircular canal detached from the utricle. Although most of the patients suffering from BPPV can be treated with correct repositioning maneuvers (1–3), some patients will still suffer from residual symptoms, including undetermined dizziness or imbalance even after typical vertigo and nystagmus disappear (4, 5). With a reported prevalence ranging from 30% to 77%, these residual symptoms have been defined as residual dizziness (RD). Residual dizziness can last from several days to a few months, adversely affecting the physical and mental health of patients (4, 5). There are currently no established diagnostic criteria for RD. Several risk factors have been reported to be associated with RD, including the long duration of BPPV before treatment (6–8), multiple repositioning maneuvers (9), elderly onset age (9, 10), physical and psychological comorbidities (9, 10), and an abnormality of vestibular evoked myogenic potential (11).

In terms of biomarker research in blood, many studies have indicated that low serum 25-hydroxyvitamin D (25(OH)D) values are related to both the occurrence and recurrence of BPPV (12, 13). Talaat et al. (12, 13) found that low values of 25(OH)D were associated with the development of BPPV, while very low 25(OH)D values were related to the recurrence of BPPV. Ding et al. (13) indicated that 25(OH)D deficiency was correlated with BPPV occurrence and recurrence, and it was also negatively associated with the severity of BPPV. Even though accumulating evidence has revealed that the low 25(OH)D level in serum increases the odds of BPPV occurrence and recurrence, whether it also contributes to RD still needs further exploration. Currently, only one study explored its effect on RD in 123 patients with posterior semicircular canal BPPV and found that low vitamin D levels were also associated with RD after successful treatment of BPPV (14). Since there were sex- and age-related differences in the vitamin D level in BPPV (15), whether these factors may influence the effect of vitamin D level on RD merits investigation.

Consequently, the present prospective short-term follow-up study was conducted among patients with BPPV after successful repositioning maneuvers, so as to explore the correlation between baseline serum 25(OH)D levels stratified by sex and onset age and RD severity 1 week later to facilitate the precise evaluation and management of RD.

## Materials and methods

### Subjects

**Figure 1:**
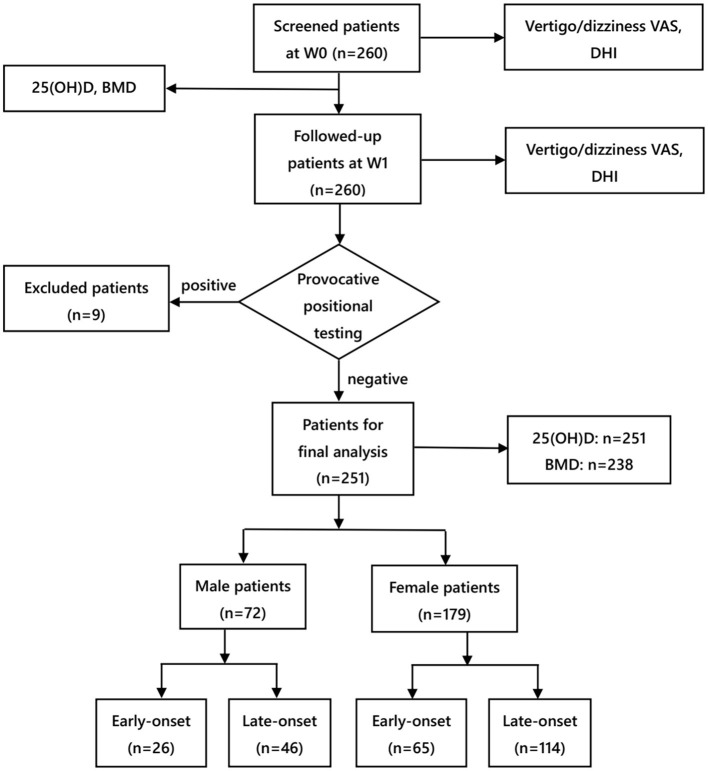
Flow chart of the study. VAS, visual analog scale; DHI, dizziness handicap inventory; BMD, bone mineral density; W0, at enrollment; W1, 1 week after enrollment.

Subjects in this study were recruited from the Vertigo Clinic of the Neurology Department in Shanghai Ninth People's Hospital Affiliated to Shanghai Jiao Tong University School of Medicine. In this study, idiopathic BPPV was diagnosed following the criteria of the American Academy of Otolaryngology-Head and Neck Surgery Guideline (16) and Barany Society (17). Each patient underwent corresponding repositioning maneuvers promptly after the diagnosis was made at the first visit. To be specific, patients diagnosed with posterior semicircular canal BPPV were repositioned by Epley's maneuver, while those diagnosed with horizontal semicircular canal BPPV were repositioned by Barbecue's or Gufoni's maneuver. The patient inclusion criteria were as follows: (i) patients aged 18–80 years, (ii) those with unilateral posterior semicircular canal BPPV or horizontal semicircular canal BPPV, (iii) those with the disappearance of positional vertigo and nystagmus after repositioning maneuvers, indicating the successful repositioning treatment at the initial visit (W0), and (iv) those who completed face-to-face follow-up 1 week (W1) after the initial visit since this time point was regarded as the short-term evaluation time recommended by the Chinese and American BPPV diagnosis and treatment guidelines (16, 18). Moreover, provocative positional testing was conducted at W1. The patient exclusion criteria were as follows: (i) those whose positional vertigo and nystagmus reappeared in provocative positional testing at W1, indicating the recurrence of BPPV, (ii) those with a previous history of deafness, vestibular neuritis, Meniere's disease, vestibular migraine, cerebrovascular disease, severe brain trauma, epilepsy, schizophrenia, and other serious physical and psychological diseases, and (iii)those who took calcium or vitamin D tablets in the past 1 month (11). From September 2017 to August 2021, altogether 260 patients with BPPV were screened, and among them, nine were excluded for disease recurrence within 1 week, leaving 251 enrolled for final analysis (Figure 1).

### Clinical profiles

Clinical parameters of all patients were collected at W0, including age, sex, body mass index (BMI), vascular comorbidities (such as hypertension, diabetes, coronary heart disease, and hyperlipidemia), history of headache, affected canals, duration of vertigo before treatment, and frequency of repositioning maneuvers. Most of our enrolled patients with BPPV were women, so the effect of estrogen on the pathogenesis of BPPV (15, 19) was also considered. According to the average menopausal age of Chinese women (20), patients aged ≤50 years were defined as the early-onset subgroup, whereas those aged >50 years were defined as the late-onset subgroup.

In this study, vertigo visual analog scale (VAS) and dizziness VAS were utilized to evaluate the severity of RD (21). The scores ranged from 0 (no symptoms) to 10 (very severe symptoms), representing the different symptom severity. Residual dizziness is a relatively subjective manifestation, and there are few relevant studies concerning the quantitative evaluation of RD. According to the principles in pain research and our previous study on BPPV (11, 22), RD was defined by the following criteria: (i) patients still suffered from dizziness or imbalanced symptoms without positional vertigo or nystagmus, as determined by provocative positional testing at W1 and (ii) the dizziness VAS score was more than 1 point at W1. Furthermore, patients with a dizziness VAS score of 1–3 were classified into a minor RD group (Group B), while those with a dizziness VAS score of >3 were in the moderate-to-severe RD group (Group C). Moreover, patients with a dizziness VAS score of 0 were classified into the no RD group (Group A). Furthermore, the Dizziness Handicap Inventory (DHI) questionnaire was used to assess the quality-of-life status of the patients. It covers three subdomains, namely, physical (DHI-P), functional (DHI-F), and emotional (DHI-E), with the total score ranging from 0 to 100 points (11, 23). A higher score indicates a greater impact on a patient's quality of life (24). DHI total and subdomain scores, together with vertigo/dizziness VAS, were evaluated and recorded at both W0 and W1 (Figure 1).

Venous blood was collected from each enrolled patient after repositioning maneuvers at W0. In our previous study, the serum 25(OH)D levels were determined by a chemiluminescence immunoassay method (Diasorin Inc, USA)(25). For the evaluation of bone mineral density, T scores of the lumbar spine and femur were measured by dual X-ray absorptiometry (GE Lunar iDXA). Osteoporosis was determined when the T score was ≤ −2.5 in either location (26).

### Statistical analysis

Statistical analysis was performed by the SPSS 26.0 software (version 26.0 for Windows). Diagrams were plotted with GraphPad Prism 5.0 for Windows. Mean and standard deviation were used to represent numerical variables with a normal distribution, whereas the median and interquartile range (IQR, 25th−75th percentile) were employed to describe numerical variables with skewed distributions. The F-test and *t-*test were adopted to compare variables with normal distribution, whereas the Kruskal–Wallis test was employed to compare variables with skewed distributions. Meanwhile, the chi-square test or Fisher's exact test was applied to categorical variables. The independent factors associated with RD were investigated by binary logistic regressions. A *P*-value of <0.05 was considered statistically significant.

## Results

### Demographic data and RD incidence

The ages of our enrolled patients were between 21 and 80 (average, 55.2 ± 14.3) years. There were 91 patients aged under 50 years in the early-onset subgroup, and 160 aged over 50 years in the late-onset subgroup. Moreover, there were 179 (65 early- and 114 late-onset) female patients and 72 (26 early- and 46 late-onset) male patients in total. At W1, 131 patients developed RD, resulting in an RD prevalence of 52.2%, including 29.1% (*n* = 73) of minor RD while 23.1% (*n* = 58) of moderate-to-severe RD.

### Serum 25(OH)D values correlated with age and sex

**Figure 2:**
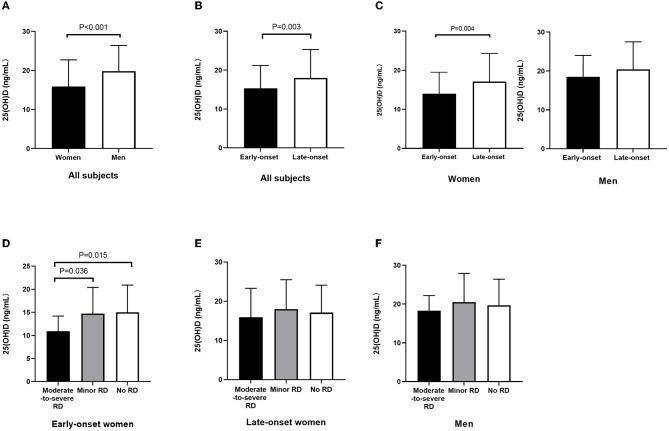
Serum 25(OH)D level among subgroups stratified by sex and onset age. **(A)** Decreased 25(OH)D level in female patients relative to male patients (*p* < 0.001). **(B)** Decreased 25(OH)D level in early-onset relative to late-onset cases (*p* = 0.003). **(C)** Decreased 25(OH)D level in early-onset female patients relative to late-onset female patients (*p* = 0.004); 25(OH)D level was not significantly different between early- and late-onset male patients. **(D)** Among early-onset female patients, 25(OH)D level in patients with moderate-to-severe RD decreased relative to those in the minor RD group (*p* = 0.036) and no RD group (*p* = 0.015). **(E)** Among late-onset female patients, the 25(OH)D level did not exhibit any significant difference among the three groups stratified by RD severity. **(F)** Among male patients, the 25(OH)D level did not exhibit any significant difference among the three groups stratified by RD severity. 25(OH)D, 25-hydroxyvitamin D; RD, residual dizziness.

The serum 25(OH)D level in female patients significantly decreased compared to those in male patients (15.9 ± 6.8 vs. 19.8 ± 6.6 ng/ml, *p* < 0.001) (Figure 2A). In addition, the 25(OH)D level decreased in early-onset patients relative to late-onset patients (15.3 ± 5.9 vs. 18.0 ± 7.3 ng/ml, *p* = 0.003) (Figure 2B). Furthermore, early-onset female patients had a lower 25(OH)D level than late-onset female patients (14.0 ± 5.5 vs. 17.1 ± 7.2 ng/ml, *p* = 0.004) (Figure 2C). Since the 25(OH)D level was not significantly different between early- and late-onset male patients (18.5 ± 5.5 vs. 20.4 ± 7.1ng/ml, *p* = 0.248) (Figure 2C), the early- and late-onset male patients were integrated into one entity. The serum 25(OH)D level in early-onset female patients decreased compared with late-onset female and male patients (14.0 ± 5.5 vs. 17.1 ± 7.2 vs. 19.7 ± 6.6 ng/ml, *p* < 0.001) (**Table 5**).

### Serum 25(OH)D values correlated with RD severity in the early-onset female subgroup

Among the early-onset female patients, the serum 25(OH)D level in the moderate-to-severe RD group decreased relative to the minor RD group (10.9 ± 3.3 vs. 14.7 ± 5.7 ng/ml, *p* = 0.036) and no RD group (10.9 ± 3.3 vs. 15.0 ± 5.9 ng/ml, *p* = 0.015). Furthermore, the serum 25(OH)D level decreased significantly in the moderate-to-severe RD group, and there was a significant difference among the three groups (*p* = 0.046). (Figure 2D, Table 1). As for late-onset female and male patients, the 25(OH)D level did not exhibit any significant difference among the three groups stratified by RD severity (Figures 2E, F, Tables 2, 3). Logistic regression analysis indicated that the low serum 25(OH)D level was closely related to the occurrence of moderate-to-severe RD in the early-onset female subgroup (OR = 0.801, 95% CI: 0.662–0.969; *p* = 0.022) (Table 4).

**Table 1:** Serum 25(OH)D level and clinical characteristics in early-onset female patients stratified by RD severity.

**Variable**	**No RD group A**	**Minor RD group B**	**Moderate- to-severe RD group C**	***p-*** **value**
Number, *n*	35	15	15	
Age (year)	38.9 ± 8.8	39.1 ± 8.4	38.4 ± 9.6	0.976
25 (OH)D (ng/mL)	15.0 ± 5.9	14.7 ± 5.7	10.9 ± 3.3	**0.046** ^*^
BMI (kg/m2)	22.2 ± 3.1	22.3 ± 2.0	19.8 ± 2.0	**0.012** ^**^
**Involved canal**, ***n*** **(%)**				0.772
Posterior	22 (51.2)	10 (23.2)	11 (25.6)	
Horizontal	13 (59.1)	5 (22.7)	4 (18.2)	
**CRPs**, ***n*** **(%)**				0.303
Multiple	12 (54.6)	7 (31.8)	3 (13.6)	
Single	23 (53.5)	8 (18.6)	12 (27.9)	
Vertigo duration (days)	7 (3–10)	7 (3.5–10)	7 (2–17.5)	0.954
With vascular comorbidities, *n* (%)	4 (50.0)	3 (37.5)	1 (12.5)	0.525
Without vascular comorbidities, *n* (%)	31 (54.4)	12 (21.0)	14 (24.6)	
With headache, *n* (%)	8 (36.4)	6 (27.2)	8 (36.4)	0.096
Without headache, *n* (%)	27 (62.8)	9 (20.9)	7 (16.3)	
With osteoporosis, *n* (%)	1 (50.0)	0 (0.0)	1 (50.0)	0.420
Without osteoporosis, *n* (%)	31 (54.4)	14 (24.6)	12 (21.0)	
**W0**				
DHI total score	28 (14–41)	36 (23–46)	46 (34–52)	0.059
DHI-P	10 (5–15)	11 (8–17)	18 (10–18)	0.183
DHI-F	14 (7–19)	19 (13–23)	14 (14–22)	0.253
DHI-E	4 (2–8)	5 (1–14)	10 (6–14)	0.110
DHI total score >30, *n* (%)	12 (40.0)	8 (26.7)	10 (33.3)	0.086
DHI total score ≤ 30, *n* (%)	17 (68.0)	5 (20.0)	3 (12.0)	
Vertigo VAS	9 (7–10)	9 (8–10)	9 (8–9)	0.840
Dizziness VAS	3 (0–6)	5 (2–8)	6 (3–7)	0.233
**W1**				
DHI total score	1 (0–4)	6 (2–10)	16 (12–30)	**< 0.001** ^***^

25 (OH)D, 25-hydroxyvitamin D; RD, residual dizziness; BMI, body mass index; DHI, dizziness handicap inventory; DHI-P, DHI-physical subdomain; DHI-F, DHI-functional subdomain; DHI-E, DHI-emotional subdomain; W0, at enrollment; W1, 1 week after enrollment; VAS, visual analog scale;

* *p* < 0.05;

** *p* < 0.01;

*** *p* < 0.001.

Statistically significant *p*-values were marked in bold.

**Table 2:** Serum 25 (OH)D level and clinical characteristics in late-onset female patients stratified by RD severity.

**Variable**	**No RD group A**	**Minor RD group B**	**Moderate- to-severe RD group C**	***p-*** **value**
Number, *n*	49	34	31	
Age (year)	64.9 ± 8.5	64.6 ± 6.7	63.3 ± 7.3	0.671
25 (OH)D (ng/mL)	17.1 ± 7.0	18.0 ± 7.5	15.9 ± 7.4	0.531
BMI (kg/m2)	23.1 ± 4.0	22.9 ± 3.3	23.5 ± 3.1	0.814
**Involved canal**, ***n*** **(%)**				0.942
Posterior	38 (43.7)	26 (29.9)	23 (26.4)	
Horizontal	11 (40.8)	8 (29.6)	8 (29.6)	
**CRPs**, ***n*** **(%)**				0.156
Multiple	16 (38.1)	17 (40.5)	9 (21.4)	
Single	33 (45.8)	17 (23.6)	22 (30.6)	
Vertigo duration (days)	7 (3.5–14)	14 (7–21)	7 (6–30)	0.101
With vascular comorbidities, *n* (%)	25 (39.7)	20 (31.7)	18 (28.6)	0.730
Without vascular comorbidities, *n* (%)	24 (47.1)	14 (27.4)	13 (25.5)	
With headache, *n* (%)	12 (41.4)	8 (27.6)	9 (31.0)	0.861
Without headache, *n* (%)	37 (43.5)	26 (30.6)	22 (25.9)	
With osteoporosis, *n* (%)	14 (41.2)	10 (29.4)	10 (29.4)	0.953
Without osteoporosis, *n* (%)	34 (43.0)	24 (30.4)	21 (26.6)	
**W0**				
DHI total score	32 (20–50)	36 (21–42)	43 (33–53)	0.268
DHI-P	11 (8–16)	12 (8–16)	16 (11–20)	0.053
DHI-F	16 (10–24)	14 (8–21)	23 (18–27)	**0.010** ^*^
DHI-E	5 (2–10)	4 (1–9)	4 (2–14)	0.774
DHI total score >30, *n* (%)	26 (40.6)	19 (29.7)	19 (29.7)	0.715
DHI total score ≤ 30, *n* (%)	19 (43.2)	15 (34.1)	10 (22.7)	
Vertigo VAS	9 (7–10)	9 (7–10)	10 (8–10)	0.269
Dizziness VAS	3 (0–6)	4 (1–5)	4 (2–6)	0.760
**W1**				
DHI total score	0 (0–4)	10 (6–22)	24 (13–46)	**< 0.001** ^***^

25 (OH)D, 25-hydroxyvitamin D; RD, residual dizziness; BMI, body mass index; DHI, dizziness handicap inventory; DHI-P, DHI-physical subdomain; DHI-F, DHI-functional subdomain; DHI-E, DHI-emotional subdomain; W0, at enrollment; W1, 1 week after enrollment; VAS, visual analog scale.

* *p* < 0.05;

^**^p < 0.01;

*** *p* < 0.001.

Statistically significant *p*-values were marked in bold.

**Table 3:** Serum 25 (OH)D level and clinical characteristics in male patients stratified by RD severity.

**Variable**	**No RD group A**	**Minor RD group B**	**Moderate- to-severe RD group C**	***p-*** **value**
Number, *n*	36	24	12	
Age (year)	55.3 ± 15.1	54.9 ± 12.5	57.3 ± 9.4	0.876
25(OH)D, (ng/mL)	19.7 ± 6.7	20.5 ± 7.4	18.3 ± 3.9	0.661
BMI (kg/m2)	24.6 ± 2.8	25.0 ± 2.9	24.9 ± 2.7	0.878
**Involved canal**, ***n*** **(%)**				0.680
Posterior	29 (52.7)	17 (30.9)	9 (16.4)	
Horizontal	7 (41.2)	7 (41.2)	3 (17.6)	
**CRPs**, ***n*** **(%)**				0.461
Multiple	12 (48.0)	7 (28.0)	6 (24.0)	
Single	23 (50.0)	17 (37.0)	6 (13.0)	
Vertigo duration (days)	7 (5–14)	4 (2–7.5)	7 (4.5–25.5)	**0.009** ^**^
With vascular comorbidities, *n* (%)	13 (40.6)	13 (40.6)	6 (18.8)	0.353
Without vascular comorbidities, *n* (%)	23 (57.5)	11 (27.5)	6 (15.0)	
With headache, *n* (%)	5 (45.5)	5 (45.5)	1 (9.0)	0.585
Without headache, *n* (%)	31 (50.8)	19 (31.2)	11 (18.0)	
With osteoporosis, *n* (%)	5 (50.0)	3 (30.0)	2 (20.0)	0.898
Without osteoporosis, *n* (%)	30 (53.6)	18 (32.1)	8 (14.3)	
**W0**				
DHI total score	22 (15–30)	26 (16–32)	36 (18–44)	**0.027** ^*^
DHI-P	8 (6–13)	8 (4–12)	12 (8–16)	0.223
DHI-F	12 (6–14)	13 (8–16)	16 (10–22)	0.243
DHI-E	2 (0–6)	4 (0–6)	6 (0–12)	0.382
DHI total score >30, *n* (%)	8 (33.3)	8 (33.3)	8 (33.3)	**0.018** ^*^
DHI total score ≤ 30, *n* (%)	28 (58.4)	16 (33.3)	4 (8.3)	
Vertigo VAS	8 (6–9)	9 (7–10)	9 (7–10)	0.194
Dizziness VAS	3 (1–5)	2 (0–3)	3 (3–7)	0.051
**W1**				
DHI total score	0 (0–4)	8 (4–10)	15 (10–33)	**<0.001** ^***^

Notes: 25 (OH)D, 25-hydroxyvitamin D; RD, residual dizziness; BMI, body mass index; DHI, dizziness handicap inventory; DHI-P, DHI-physical subdomain; DHI-F, DHI-functional subdomain; DHI-E, DHI-emotional subdomain; W0, at enrollment; W1, 1 week after enrollment; VAS, visual analog scale.

* *p* < 0.05;

** *p* < 0.01;

*** *p* < 0.001.

Statistically significant *p*-values were marked in bold.

**Table 4:** Logistic regression analyses for moderate-to-severe RD in different subgroups of patients with BPPV.

**Group**	**β**	**OR**	**95%CI**	***p-*** **value**
**Early-onset female patients (Group C vs. Group A)**				
25 (OH)D	−0.222	0.801	0.662–0.969	**0.022** ^*^
**Late-onset female patients (Group C vs. Group A)**				
DHI-F at W0	0.067	1.069	1.002–1.140	**0.044** ^*^
**Male patients (Group C vs. Group A)**				
DHI total score>30 at W0	2.082	8.019	1.745–36.848	**0.007** ^**^

BPPV, benign paroxysmal positional vertigo; RD, residual dizziness; Group C represents patients with moderate-to-severe RD; Group A represents patients with no RD; 25 (OH)D, 25-hydroxyvitamin D; DHI, dizziness handicap inventory; DHI-F, DHI-functional subdomain; W0, at enrollment; W1, 1 week after enrollment; CI, confidence interval.

* *p* < 0.05;

** *p* < 0.01.

Statistically significant *p*-values were marked in bold.

### Clinical characteristics associated with RD severity in late-onset female and male subgroups

Among the late-onset female patients, DHI-F scores were closely related to the RD severity (*p* = 0.010) (Table 2). Based on logistic regression analysis, the higher DHI-F scores at W0 increased the risk of moderate-to-severe RD in this subgroup (OR = 1.069, 95% CI: 1.002–1.140; *p* = 0.044) (Table 4). With regard to the male subgroup, DHI total scores (*p* = 0.027) and duration before treatment (*p* = 0.009) were associated with RD severity (Table 3). According to logistic regression analysis, DHI total scores of >30 at W0 were related to the occurrence of moderate-to-severe RD in this subgroup (OR = 8.019, 95% CI: 1.745–36.848; *p* = 0.007) (Table 4).

### Other clinical findings

Compared with the other two subgroups, the late-onset female subgroup exhibited the highest proportion of osteoporosis (*p* < 0.001), vascular comorbidities (*p* < 0.001), longest vertigo duration (*p* = 0.007), highest DHI total scores (*p* = 0.001) and three subdomain scores (*p* < 0.01) at W0. By contrast, the early-onset female subgroup had the highest proportion of headaches (*p* = 0.041) (Table 5).

**Table 5:** Serum 25 (OH)D level and clinical characteristics among different subgroups of patients with BPPV.

**Variable**	**Early-onset female patients**	**Late-onset female patients**	**Male patients**	***p-*** **value**
Number, *n*	65	114	72	
Age (year)	38.8 ± 8.8	64.4 ± 7.6	55.5 ± 13.3	**< 0.001** ^***^
25 (OH)D, (ng/mL)	14.0 ± 5.5	17.1 ± 7.2	19.7 ± 6.6	**< 0.001** ^***^
BMI (kg/m^2^)	21.7 ± 2.8	23.1 ± 3.6	24.8 ± 2.8	**< 0.001** ^***^
Vertigo duration (days)	7 (3–12.5)	9 (5–21)	7 (3.5–14)	**0.007** ^**^
**Involved canal**, ***n*** **(%)**				0.275
Posterior	43 (23.3)	87 (47.0)	55 (29.7)	
Horizontal	22 (33.3)	27 (40.9)	17 (25.8)	
**CRPs**, ***n*** **(%)**				0.919
Multiple	22 (24.7)	42 (47.2)	25 (28.1)	
Single	43 (26.7)	72 (44.7)	46 (28.6)	
With vascular comorbidities, *n* (%)	8 (7.8)	63 (61.1)	32 (31.1)	**< 0.001** ^***^
Without vascular comorbidities, *n* (%)	57 (38.5)	51 (34.5)	40 (27.0)	
With headache, *n* (%)	22 (35.5)	29 (46.8)	11 (17.7)	**0.041** ^*^
Without headache, *n* (%)	43 (22.7)	85 (45.0)	61 (32.3)	
With osteoporosis, *n* (%)	2 (4.4)	34 (73.9)	10 (21.7)	**< 0.001** ^***^
Without osteoporosis, *n* (%)	57 (29.7)	79 (41.1)	56 (29.2)	
**W0**				
DHI total score	34 (22–46)	36 (26–48)	24 (16–36)	**0.001** ^**^
DHI-P	11 (8–18)	12 (8–16)	8 (6–13)	**0.003** ^**^
DHI-F	16 (11–22)	18 (10–24)	12 (7–16)	**< 0.001** ^***^
DHI-E	6 (2–12)	4 (2–10)	2 (0–8)	**0.007** ^**^
DHI total score>30, *n* (%)	29 (24.8)	64 (54.7)	24 (20.5)	**0.002** ^**^
DHI total score ≤ 30, *n* (%)	25 (21.4)	44 (37.6)	48 (41.0)	
Vertigo VAS	9 (7–10)	9 (8–10)	9 (7–10)	**0.041** ^*^
Dizziness VAS	5 (2–6)	4 (1–6)	3 (1–5)	0.080
**W1**				
DHI total score	6 (0–14)	8 (0–22)	4 (0–10)	0.053
Dizziness VAS	1 (0–3)	2 (0–4)	1 (0–3)	0.257
**RD**				
No RD, *n* (%)	35 (29.2)	49 (40.8)	36 (30.0)	0.341
Minor RD, *n* (%)	15 (20.5)	34 (46.6)	24 (32.9)	
Moderate-to-severe RD, *n* (%)	15 (25.9)	31 (53.4)	12 (20.7)	

BPPV, benign paroxysmal positional vertigo; 25 (OH)D, 25-hydroxyvitamin D; RD, residual dizziness; BMI, body mass index; DHI, dizziness handicap inventory; DHI-P, DHI-physical subdomain; DHI-F, DHI-functional subdomain; DHI-E, DHI-emotional subdomain; W0, at enrollment; W1, 1 week after enrollment; VAS, visual analog scale.

* *p* < 0.05;

** *p* < 0.01;

*** *p* < 0.001.

Statistically significant *p*-values were marked in bold.

## Discussion

Residual dizziness is a common complaint by patients with BPPV, even after successful repositioning. This short-term follow-up study was conducted on 251 patients, aiming to analyze the correlation between baseline serum 25(OH)D levels and RD severity 1 week after successful repositioning maneuvers. Our results revealed that there were differences in serum 25(OH)D levels among patients with regard to sex and onset age, and early-onset female patients had the lowest 25(OH)D level relative to late-onset female and male patients. Moreover, in the subgroup of early-onset female patients, the decreased 25(OH)D level increased the risk of moderate-to-severe RD 1 week after successful repositioning maneuvers.

Interestingly, it was found that compared with late-onset patients with BPPV, the 25(OH)D level decreased in early-onset patients, with early-onset female patients having the lowest 25(OH)D level, which was consistent with other studies on vitamin D levels in Asian populations (15, 27, 28). Serum 25(OH)D levels may be affected by several factors, such as age, sex, seasonality, hormonal factors, geographic location, nutrition, lifestyle habits, and preexisting metabolic disorders. As demonstrated in a systematic review (27), in the Asia/Pacific region, 25(OH)D levels in children and adolescents are significantly lower than those in adults and elderly people. Han et al. (28) observed a significant association between 25(OH)D and increasing age, and the older individuals had higher serum 25(OH)D concentrations than the younger ones. Furthermore, Song et al. (15) also found that vitamin D levels varied with sex and age. Serum 25(OH)D level in female patients was significantly lower than those in male patients, and their level gradually increased with age in both BPPV and control groups. Solar radiation remains the most important source of vitamin D in human beings (29, 30), while serum 25(OH)D is the main circulating form of vitamin D, which is commonly used to assess the vitamin D status in a patient (29–31). The main cause of vitamin D deficiency in early-onset female patients in our study remains unknown, which may be attributed to inadequate exposure to sunlight due to the lack of outdoor activity, the use of sunscreen, and increased physiological demands, such as pregnancy or lactation (29, 31).

Previous studies have suggested the following possible mechanism of RD: (i) the persistence of otoconial debris in the semicircular canal, insufficient to provoke cupula deflection, leads to slight vertigo without positional nystagmus; (ii) a utricular dysfunction accompanying BPPV or an undiagnosed coexisting vestibular disorder; (iii) an incomplete or delayed central adaptation after repositioning maneuvers; (iv) sympathoneural dysregulation when the symptoms of RD are similar to those of autonomic dysfunction; and (v) emotional factors, anxiety developed after acute vertigo (4, 6–8). There is little relevant literature on whether 25(OH)D deficiency is associated with RD. Wu et al. (14) recently reported that low 25(OH)D levels are associated with the development of RD in patients with PC-BPPV. In our study, we quantified and stratified the severity of RD by VAS. A previous investigation indicated that VAS exhibits higher sensitivity and specificity in RD assessment (32). For RD, we have detailed severity evaluation and concrete evaluation time. In addition to 25(OH)D, more potential clinical factors associated with RD, such as vascular comorbidities, osteoporosis, and affected canals, were analyzed. The effect of vitamin D on RD was further stratified by sex and onset age in our study. A novel finding was that the serum 25(OH)D level was related to the severity of RD in early-onset female patients, and the low 25(OH)D level increased the risk of moderate-to-severe RD in this subgroup, but this effect was not found in late-onset female and male patients.

One of the main pathophysiological mechanisms of RD may be related to the existence of residual small otoconial debris in the semicircular canal after repositioning maneuvers, which is insufficient to provoke cupula deflection, causing mild dizziness or imbalance (4–7). Physiologically, 1, 25-dihydroxyvitamin D, the biologically active form of 25(OH)D, is beneficial for the upregulation of epithelial Ca^2+^ channel transporters, such as ECaC1, calbindin-D9k, and calbindin-D28k, in the semicircular canal, which can maintain the low endolymph Ca^2+^ concentration in the inner ear. Notably, a low endolymph Ca^2+^ concentration is significant for preventing the production of abnormal otoconia and maintaining the capacity to dissolve exfoliated otoconia. Therefore, a low vitamin D level can prompt the formation of otoconia or disturb the resorption of detached otoconia in endolymph (15, 33, 34). In addition, the physiological function of vitamin D depends on vitamin D receptors (VDR) in the inner ear epithelial cells. Vitamin D/VDR plays a very important role in vestibular function, and VDR-deficient mice show a decreased equilibrium function, suggesting that vitamin D deficiency may lead to vestibular dysfunction (13, 35). Therefore, it is inferred that vitamin D deficiency may cause the residues of small otoconial debris and additional dysfunction of otolith organs, contributing to the development of moderate-to-severe RD.

In late-onset female and male patients whose serum 25(OH)D levels were comparatively higher, vitamin D had an insignificant impact on RD severity. Late-onset female and male patients had higher proportions of vascular comorbidities and osteoporosis. As reported in a multicenter observational study, the presence of comorbidities such as hypertension, diabetes, and osteoporosis might worsen the status of the labyrinth, leading to more common otolith detachment and increasing the risk of BPPV recurrence (36). However, there is no clear conclusion about their correlation with RD, and the correlation between RD severity and comorbidities was not found in this study.

Instead, in this study, the higher DHI-F scores and DHI total scores at the baseline were related to the occurrence of moderate-to-severe RD 1 week later in late-onset female and male subgroups, respectively. Previous studies have revealed that the total DHI scores, especially DHI-F and DHI-E scores at the baseline, are significantly higher in the RD group than in the non-RD group. This indicates that patients with BPPV who have their life and emotional state more greatly affected before treatment are more likely to develop RD (37, 38). Fu et al. (39) found that patients with a DHI score of >30 had a nearly 2-fold higher risk of RD compared to those with a DHI score of ≤30. Previous investigations have found a strong correlation between DHI scores and comorbid anxiety and depression (40). Compared to early-onset female and male patients, late-onset female patients exhibited higher DHI scores at the baseline, indicating that this subgroup tended to have more serious mental health impairments. Cetin et al. (41) observed a correlation between RD and high anxiety levels in elderly patients with BPPV. Moreover, late-onset female patients had the longest vertigo duration. Furthermore, Faralli et al. (8) found that patients with BPPV who had longer durations before treatment tended to have higher DHI scores and a higher proportion of RD. The BPPV-induced peripheral vestibular asymmetry can lead to new central adaptations. The longer duration of otoconia debris in the endolymph will induce a better-established adaptation. Despite successful treatment, the brain cannot rapidly readapt to the pattern after repositioning maneuvers, resulting in more severe RD (8). It was inferred in this study that patients with higher DHI scores potentially had delayed central readaptation, which might contribute to persistent RD. An rs-fMRI study from our team found pontine functional alterations in patients with BPPV, which might reflect the central adaptation of RD (42). Further neuroimaging studies are warranted to explore the neural basis of RD in BPPV patients.

Our study has important clinical implications. Residual dizziness in BPPV may have varying etiology in patients of different sex and onset age, and a personalized treatment strategy is needed. To be specific, for early-onset female patients with low serum 25(OH)D level, the otolith organ disorder might be the most probable cause of RD, thus, the supplementation of vitamin D is necessary, and more outdoor activities and sun exposure are encouraged. For late-onset female patients, delayed central adaptation and comorbid anxiety and depression might be the most likely cause of RD, so timely diagnosis and treatment are particularly important for elderly patients with BPPV, and high attention should be paid to their potential psychological problems. Nonetheless, certain limitations should be noted in this study. First, the sample size of our study was relatively limited. Second, vitamin D data in healthy controls were not available, and several immune-related indicators, such as serum thyroid antibodies and rheumatoid factor, also may influence vitamin D expression. Further studies should be undertaken to correct these confounding factors. Third, the follow-up period was relatively short. Therefore, further follow-up investigations are required to reveal the role of vitamin D deficiency in the long-term RD of BPPV.

## Conclusion

There were differences in serum 25(OH)D levels among patients with BPPV with regard to age and sex, and early-onset female patients had lower 25(OH)D levels than late-onset female and male patients. Decreased 25(OH)D level in early-onset female patients may increase the odds of short-term moderate-to-severe RD after successful repositioning maneuvers. In future, further follow-up study is warranted to disclose the effect of vitamin D deficiency on long-term RD of BPPV.

## Data availability statement

The original contributions presented in the study are included in the article/supplementary material, further inquiries can be directed to the corresponding authors.

## Ethics statement

The studies involving human participants were reviewed and approved by the Ethics Committee of Shanghai Ninth People's Hospital Affiliated to Shanghai Jiao Tong University School of Medicine (ethical approval number: 2018-128-T106). The patients/participants provided their written informed consent to participate in this study.

## Author contributions

JW contributed to the provision of study materials, data collection, analysis, and interpretation. C-YJ and Y-XB contributed to the collection and assembly of data, data analysis, and interpretation. QX contributed to the collection and assembly of data. X-HS, HP, and LS contributed to the provision of study materials. J-RL made important contributions to administrative support, data analysis, and interpretation. WC made important contributions to the conception, design, provision of study materials, data analysis, and interpretation. All authors contributed to the writing and approved the final version of the manuscript.
